# Facilitators and barriers to community-based HIV testing in Guinea: a CFIR-based implementation analysis

**DOI:** 10.3389/fpubh.2025.1593697

**Published:** 2025-07-24

**Authors:** Soriba Camara, Tamba Mina Millimouno, Mory 1 Kourouma, Abdoulaye Sow, Sidikiba Sidibé, Aly Badara Touré, Aly Badara Nabé, Alexandre Delamou

**Affiliations:** ^1^Department of Public Health, Faculty of Health Sciences and Techniques, Gamal Abdel Nasser University of Conakry, Conakry, Guinea; ^2^Africa Center of Excellence for Prevention and Control of Communicable Diseases (CEA-PCMT), Gamal Abdel Nasser University of Conakry, Conakry, Guinea; ^3^Guinea Infectious Disease Research and Training Center, Gamal Abdel Nasser University of Conakry, Conakry, Guinea

**Keywords:** community-based testing, facilitators, barriers, HIV, CFIR, index case, Guinea

## Abstract

**Introduction:**

In Guinea, where 36% of people living with HIV (PLHIV) are unaware of their serostatus, innovative screening strategies are crucial to achieving the joint United Nations Program on HIV/AIDS’ 95–95-95 targets. Community-based HIV testing, as recommended by the World Health Organization, aims to reach at-risk populations by leveraging local resources and actors. Using the Consolidated Framework for Implementation Research (CFIR), this study assessed facilitators and barriers to implementing community-based HIV screening across 10 pilot sites in Guinea, with the goal to optimizing its effectiveness.

**Methods:**

This qualitative descriptive study applied the CFIR framework to identify factors influencing the implementation of community-based HIV screening and capture the nuanced perspectives of stakeholders. Overall, 28 in-depth interviews were conducted with key participants, including PLHIV, health workers, community-based actors, and members of the national coordination teams.

**Results:**

Home-based testing was identified as a key facilitator for improving access to healthcare by reducing financial and logistical barriers. However, several barriers hindered its effectiveness, including frequent stock shortages, concerns about confidentiality and stigma, insufficient training and incentives for community counselors, and the absence of clear protocols defining the roles and responsibilities of stakeholders.

**Conclusion:**

The findings emphasize the need to strengthen community-based HIV testing in Guinea by ensuring a consistent supply of essential resources, enhancing coordination among stakeholders, and providing adequate incentives for community counselors. Integrating this approach into national policies could enhance both its effectiveness and sustainability, offering actionable insights for adapting HIV testing strategies in similar resource-limited settings.

## Introduction

Despite notable progress in fighting HIV, the epidemic remains a major global public health challenge. Approximately 10 million people still lack access to antiretroviral therapy (ART), and nearly 48% of infants living with HIV do not receive essential care (1, 2). These disparities jeopardize the achievement of the “95–95-95” targets, which aim to diagnose 95% of people living with HIV, treat 95% of those diagnosed, and achieve viral suppression in 95% of those on therapy by 2030 (1, 3). In low-income countries, socioeconomic inequalities and persistent stigma continue to restrict access to HIV testing and treatment services (4, 5). Since 1994, the World Health Organization has emphasized that integrating screening and counseling is essential for reducing transmission and promoting early detection (6–8). Studies consistently show that involving communities in health interventions improves access and uptake by leveraging local networks and engaging trusted community agents (9–16). In Guinea, where the HIV prevalence is approximately 1.5%, substantial gaps remain in access to screening and treatment. An estimated 36% of people living with HIV are unaware of their status, and mother-to-child transmission (MTCT) rates remain alarmingly high (17–19). Although antenatal care (ANC) visits provide critical opportunities for HIV testing, they often fail to effectively engage male partners and family members of HIV-positive women. Structural challenges, including frequent stockouts, poor coordination among health actors, and ongoing stigma, further exacerbate these issues. Recent studies from sub-Saharan Africa have highlighted the specific operational and psychosocial challenges of community-based HIV screening in the region, reinforcing the need for tailored, context specific (20–28).

To address these challenges, Guinea has implemented a community-based HIV screening approach targeting the partners, family members, and close contacts of HIV-positive pregnant women (index case) enrolled in prevention of mother-to-child transmission (PMTCT) services. In this context, the term “index case” refers to an HIV-positive pregnant woman enrolled in PMTCT services whose diagnosis serves as the initial reference point for targeted contact tracing. Identifying the index case enables community-based screening among her social network, thereby promoting early detection and linkage to care for those at risk (29). This intervention relies on collaboration between health centers and community-based organizations (CBOs). Trained community counselors are responsible for identifying, sensitizing, and screening the husbands and family members of HIV-positive women. They also provide psychosocial support and referrals to health services to enhance acceptability and service uptake. While community-based HIV testing has been evaluated in other African contexts (30–33), limited evidence exists regarding its implementation in Guinea, particularly using the Consolidated Framework for Implementation Research (CFIR). This study aims to assess the facilitators and barriers influencing the implementation of community-based HIV testing across 10 pilot sites in Guinea using the CFIR Framework (34). By identifying key factors that influence the implementation success, this research provides actionable recommendations to strengthen HIV screening strategies, reduce MTCT, and improve care for at-risk populations. These findings will support national health policies and guide future community-based interventions in Guinea and other resource-limited settings.

## Methods

### Study design

We conducted a descriptive qualitative study over a three-weeks period to explore the factors influencing the implementation of community-based HIV testing around index cases in pilot sites. This approach was selected for its ability to generate in-depth insights into stakeholders’ the lived experiences and uncover contextual elements shaping implementation. The CFIR was applied to structure the analysis and rigorously interpret the data (34).

### Description of the CFIR framework

The Consolidated Framework for Implementation Research (CFIR) served as the conceptual foundation for this study, offering a systematic lens for examining the factors affecting community-based HIV testing in Guinea. The framework encompasses five key domains. *The Innovation domain* focuses on evaluating the adaptability and complexity of the intervention to ensure stakeholder acceptance. *The Outer Setting* considers external influences, including national policies and availability of resource. *The Inner Setting* addresses aspects of organizational culture, structure, and the climate for implementation. *The Characteristics of Individuals domain* explores the knowledge, attitudes and motivation of those responsible for delivering the intervention. Finally, *The Implementation Process domain* assesses how planning, stakeholder engagement, execution and evaluation are carried out. CFIR’s comprehensive and multi-level structure enabled a systematic assessment of both facilitators and barriers to implementation. Its application generated actionable insights relevant to resource-limited settings. In the context of this study, the CFIR framework proved particularly well-suited for analyzing community-based HIV testing interventions, as it captures the interplay of individual, organizational, and contextual dynamics that influence implementation outcomes.

### Theory of change

**Figure 1:**
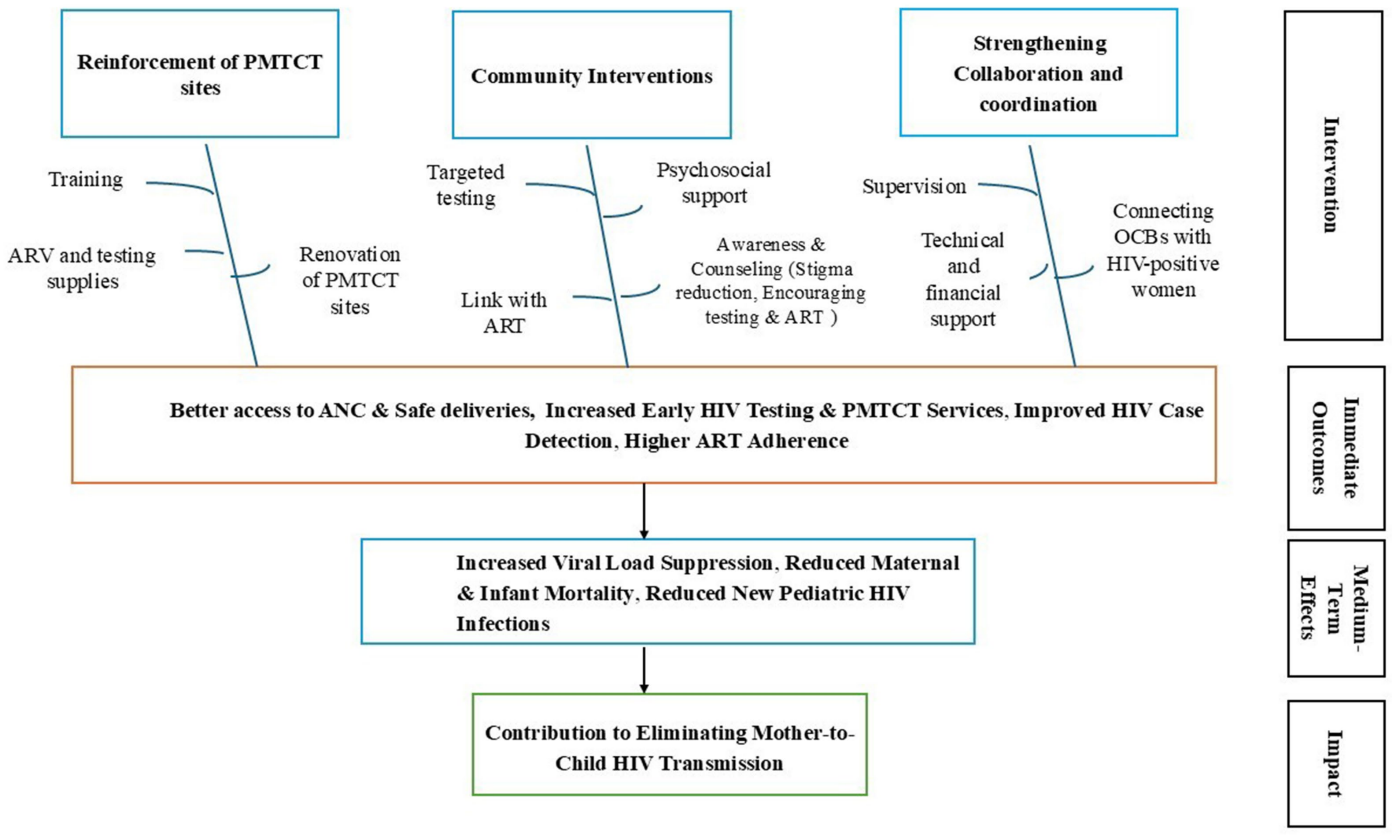
Theory of change for eliminating of mother to child HIV transmission in Guinea, December 2020–December 2024.

The pilot project on community-based HIV testing in Guinea seeks to reduce mother-to-child transmission (MTCT) of HIV through three core pillars: strengthening health services, enhancing community engagement, and fostering multi-sectoral collaboration. The first pillar, *strengthening health services*, focuses on capacity building through continuous training for healthcare workers at PMTCT-integrated health centers. It also aims to improve the supply chain by ensuring the consistent availability of HIV test kits, ARVs, and essential consumables. Additionally, infrastructure upgrades are intended to better integrate systematic HIV screening into antenatal care (ANC), thereby promoting early detection, improved PMTCT access, and safer deliveries. The second pillar, *community engagement*, emphasizes targeted HIV testing through outreach programs led by CBOs. These efforts are supported by psychosocial support and referral systems. Awareness campaigns and stigma reduction initiatives are expected to increase HIV testing uptake and adherence to treatment, ultimately fostering stronger community involvement and more effective case detection. The third pillar, strengthening multi-sectoral collaboration, promotes structured partnerships between health facilities and CBOs, alongside advocacy for policy that institutionalize community-led testing models. A robust monitoring and evaluation system will support continuous program assessment and iterative improvement. By integrating these pillars, the intervention aims to strengthen ART adherence, reduce maternal and infant mortality, and ultimately contribute to the elimination of MTCT of HIV in Guinea (Figure 1). This study assesses this innovative community-based HIV testing strategy by identifying the facilitators and barriers to its implementation in pilot sites, with the goal of optimizing its integration into national HIV control strategies.

### Study site and intervention description

The study was conducted at 10 PMTCT sites across the urban districts of N’zérékoré, Siguiri, and Boké, where a pilot community-based HIV testing initiative was implemented (2020–2023). The intervention targeted families and close contacts of HIV-positive pregnant women to enhance early detection and reduce vertical transmission. It was structured around three pillars: strengthening PMTCT-integrated health facilities, mobilizing community-based organizations (CBOs) for outreach and testing, and ensuring coordinated oversight.

Fraternité Médicale Guinée (FMG), with support from the Global Fund and in collaboration with the National HIV/AIDS Control Program, played a central role in implementation. FMG trained and mentored healthcare workers on HIV testing protocols within PMTCT services; selected and trained CBOs, supervised community counselors; supplied HIV test kits and logistics; and provided small grants, including performance-based incentives. FMG also facilitated coordination among CBOs, health facility staff, and health authorities at both national and local levels to ensure effective deployment and sustainability of the community-based testing strategy.

### Participant recruitment and data collection

A combined sampling approach was employed to ensure a diverse range of participant profiles. Purposive sampling targeted key stakeholders, including HIV-positive pregnant women, their partners, healthcare worked, representatives of CBOs, and national coordination actors. Participants were recruited through health centers, community outreach, or referrals networks. To capture varied experiences, a maximum variation strategy was applied, considering sociocultural and organizational influences. Guided by the CFIR framework, a total of 28 in-depth interviews (IDIs) were conducted by six trained researchers between December 16, 2023, and January 15, 2024. The interviews, which collectively totaled 11 h, were conducted in both French and local languages. All interviews were transcribed, anonymized, and carried out in accordance with ethical approvals. To address language barriers and enhance data accuracy, bilingual researchers were engaged during interviews conducted in local languages. This approach enabled a more nuanced and contextually rich analysis of factors of the influencing the effectiveness of community-based HIV testing.

### Data analysis

A deductive thematic analysis based on the CFIR framework structured the interpretation of data. The coding process was conducted using NVivo 14, employing a hierarchical system of codes, subcodes, and broader analytical themes. A total of five CFIR domains, 15 primary themes, and over 25 sub-nodes (barriers and facilitators) were developed to reflect both predefined constructs and emerging data. A dual coding strategy was used to ensure analytical rigor, with two independent coders achieving a Kappa coefficient of 0.90, indicating strong inter-coder reliability. Regular cross-checks and iterative discussions were conducted to resolve discrepancies and minimize bias. A codebook integrated predefined and emerging themes, refining analytical categories. Emic categories captured participants’ sociocultural perspectives and emerging themes were validated through triangulation. This approach facilitated an in-depth examination of contextual, organizational, and individual factors influencing the implementation of community-based HIV testing.

### Ethical considerations

The study protocol was approved by the Guinean National Ethics Committee for Health Research (N°066/CNERS/23). Prior to participation, an information letter was sent to the heads of pilot sites and community-based organizations, and informed consent was obtained from all participants. Data was collected anonymously to ensure confidentiality and ethical compliance throughout the study.

## Results

A total of 28 participants were included in the study, representing diverse stakeholders involved in the implementation of community-based HIV testing in Guinea. Of these, 32% were members of community-based organizations, 36% were healthcare workers, 25% were HIV-positive women receiving care at PMTCT sites, and 7% were members of the national coordination team. Nearly half of the participants (47%) were women. Participants originated from various regions and linguistic groups including Susu, Malinké, Fula, and Kpelle, allowing for a comprehensive exploration of the facilitators and barriers to the acceptability of community-based HIV testing (Table 1).

**Table 1:** Socio-demographic characteristics of participants in the community-based HIV testing study, December 2023 – January 2024 (*n* = 28).

Characteristic	Category	*n*	%
Stakeholder type	Community-based organizations	9	32
	Healthcare workers	10	36
	HIV-Positive Women (PMTCT Sites)	7	25
	National Coordination Team	2	7
Gender	Women	13	47
	Men	15	53
Region / Linguistic group	Boké	8	29
	Siguiri	7	24
	N’Zérékoré	11	39
	Conakry	2	7

### Facilitators and barriers

Qualitative analysis, guided by the CFIR framework, identified several key themes. The findings reveal that the implementation of community-based HIV testing was facilitated by factors such as the convenience of home-based testing, the emotional and social support provided by community-based organizations, and the alignment between program objectives and organizational values. However, significant barriers were also identified barriers, including frequent stockouts, inadequate training and financial incentives for community counselors, concerns about confidentiality, and coordination challenges among stakeholders (Table 2).

**Table 2:** Summary of facilitators and barriers to community-based HIV testing in Guinea, December 2023–January 2024.

Themes	CFIR domains	CFIR sub-constructs	Description	Influencing factor	Proposed solutions
Convenience	Innovation domain	Relative advantage	Home-based testing is perceived as convenient, reducing costs and time.	Facilitator	Expand home-based testing services and promote awareness campaigns to emphasize its convenience.
Social and emotional support	Innovation domain	Relative advantage	CBOs played a central role in providing emotional support to patients, particularly by encouraging their relatives to get tested.	Facilitator	Strengthen partnerships with CBOs and provide additional training to enhance their psychosocial support roles.
Confidentiality	Innovation domain	Complexity	The method is considered more confidential, although some participants fear unintentional disclosure of their HIV status.	Facilitator and Barrier	Develop strict confidentiality protocols and train counselors on maintaining privacy during home visits.
Availability of resources	Outer setting	Availability of external resources	Stockouts and a lack of clear protocols often compromise the continuity of the intervention.	Barrier	Establish an efficient supply chain management system to ensure consistent availability of testing kits and resources.
Training and motivation of community counselors	Inner setting	Incentives and rewards	Due to the lack of continuous training and adequate incentives, community counselors’ high turnover rate has affected service continuity in community-based testing.	Barrier	Implement regular training programs and provide financial and non-financial incentives to retain counselors.
Compatibility with the values of community organizations	Inner setting	Compatibility	The alignment with the values of CBOs facilitated the adoption of community-based HIV testing.	Facilitator	Foster collaboration with CBOs by aligning intervention goals with their existing values and missions.
Fear of stigmatization	Characteristics of individuals	Knowledge and beliefs	The fear of being stigmatized remains a major barrier to the acceptance of community-based HIV testing.	Barrier	Launch stigma-reduction campaigns and involve community leaders to foster acceptance of HIV testing.
Supervision and support of CBOs	Implementation process	Stakeholder engagement	Insufficient supervision and coordination and a lack of logistical support limit effectiveness.	Barrier	Develop clear operational protocols and provide logistical support to improve supervision and coordination.

#### Identified barriers

The barriers were classified according to relevant CFIR domains:

**Innovation domain**: Complexity and confidentiality

Although home-based testing is perceived as more confidential, some participants expressed concerns regarding the risk of involuntary disclosure of their HIV status. One participant stated, “*Some people fear getting tested because they worry their neighbors might discover their status*.” (CBO 4)

**Outer setting**: Resource availability

Chronic stockouts and an unreliable supply chain disrupted the continuity of community-based services. As one participant explained, *“Frequent stockouts and an uncertain supply chain limit our ability to consistently provide testing supplies.”* (CBO 6)

**Inner setting**: Coordination and motivation

The Inner Setting domain highlights several critical barriers to barriers the implementation of community-based HIV testing in Guinea. First, some health authorities express doubts about the quality of services delivered by non-medical personnel. Second, both financial and material incentives are insufficient, resulting in high turnover among community counselors and disrupting services. Finally, governance challenges, such as partial payments of stipend and demands for financial returns, undermine trust and weaken the commitment of community actors. These barriers are summarized in Table 3.

**Characteristics of individuals**: Fear of stigmatization.

**Table 3:** Summary of barriers identified in the inner setting analysis of community-based HIV testing in Guinea, December 2023 – January 2024.

Themes	Description	Verbatim	Influencing factor
Alignment of innovation with the values and objectives	Some health authorities believed that HIV testing by non-medical personnel was inappropriate.	*“They (health authorities) say blood work is for medical personnel. So, they do not agree that non-medical staff should conduct testing. “*Coordination 1	Barrier
Turnover of CBOs	High turnover among community counselors disrupted the continuity and quality of community-based testing.	*“The turnover of counsellors, due to lack of motivation, caused adaptation issues and broke the confidentiality bond with our patients, regardless of the quality of the new counsellors.” CBO 7*	Barrier
Stigmatization			
Lack of Continuous Training	The absence of ongoing training for new counselors reduced the capacity of organizations to sustain optimal skills.	*“For this approach to succeed, we must continuously train community counselors, especially new counsellors, and provide them with tools to identify cases.” CBO 1*	Barrier
Motivation Policy and Recognition	Financial incentives, including operational costs and stipends, were deemed insufficient.	*“What we receive is not enough, but I am happy because our activities help save lives.” CBO 3*	Barrier

The fear of stigma remains a major barrier to the acceptance of community-based HIV testing. One participant illustrated this reality by stating, *“When my family members are informed, I risk being rejected by everyone, and each person will interpret the information in their own way; therefore, I prefer to keep it between my husband and me.”* (Index Case 5)

**Implementation processes**: Process and coordination

Insufficient supervision, ambiguous role definitions, and inadequate logistical support delayed the distribution of supplies and hindered effective patient follow-up. As a coordinator explained, *“We sometimes revisit the same household multiple times to obtain consent, highlighting organizational challenges.”* (National Coordination 1)

To overcome institutional reluctance, Fraternité Médicale Guinée (FMG) engaged in targeted advocacy. Confronting opposition from decentralized health authorities, FMG collaborated with the National HIV/AIDS Control Program, securing a ministerial directive that endorsed community-based testing and clarified roles, ensuring better integration into the national HIV strategy (Box 1).

BOX 1 **Overcoming institutional barriers through policy advocacy for community-based HIV testing.**

*The main challenge we faced was the lack of awareness of the strategy, even among decentralized health authorities. In some districts, Health Directors refused to allow community-based organizations to conduct HIV testing because they were unaware of the strategy. I took the initiative to meet with the National HIV/AIDS Control Program, and together, we consulted the Minister of Health. The Minister then issued a circular to Health districts and centers, which significantly facilitated our work.”" (National Coordination 2)*

#### Identified facilitators

Facilitators, also organized by CFIR domains, include:

**Innovation domain**: Relative advantage and social support

Home-based testing was perceived as convenient, effectively reducing both costs and waiting times. As one participant remarked: *“With this approach, I did not have to go anywhere; the test was performed at home, without wasting time at the health center.*” (Index Case 3)

In addition, community-based organizations provide essential emotional support, which helped build trust and encouraged family members to undergo tested. One participant shared, *“When I was referred to ABEF (a CBO), they reassured me and gave me the confidence to disclose my status to my husband.”* (Index Case 6)

**Outer setting**: Financial support and resource availability

External funding, particularly through the national coordination team, strengthened operational capacity by supporting training, supplying test kits, and offering financial incentives. A participant emphasized this impact, stating, *“In terms of capacity building, we received training, testing supplies, and financial incentives.”* (CBO 4)

**Inner setting**: Alignment with community values and collaboration

The CBOs played a pivotal role in the successful implementation of community-based HIV testing. The intervention closely aligned with the values and mission of these organizations, fostering acceptance and smooth integration into community activities. A member of the coordination team stated, *“Fortunately, the CBOs we identified were already involved in community activities. Therefore, we provided them with a framework to integrate HIV-related initiatives into their mobilization efforts.”* (Coordination 1)

## Discussion

This study highlights both the facilitators and barriers that influence the implementation of community-based HIV testing in Guinea, using the CFIR framework as an analytical lens. Overall, home-based testing is well accepted due to its convenience and accessibility; however, its effectiveness is constrained by several structural challenges. These include frequent stockouts, inadequate training and financial incentives for community counselors, institutional reluctance, and weak coordination mechanisms. In addition, concerns related to confidentiality and stigma continue to hinder beneficiaries’ willingness to participate in testing.

Our findings confirm that community-based HIV testing substantially improves accessibility for hard-to-reach populations. A study conducted in rural South Africa (32, 35) similarly underscored the pivotal role of community-based organizations in delivering essential psychosocial support and guidance to newly diagnosed individuals. Moreover, our results are consistent with evidence from Uganda and Kenya, where community counselors have been shown to play a crucial role in increasing HIV testing uptake (33). This relative advantage is further reinforced by the approach’s affordability and capacity to overcome geographical and financial barriers that frequently restrict access to conventional healthcare facilities (36–38).

The importance of social support and trust in enhancing HIV testing uptake is widely recognized in the literature. The emotional support provided by CBOs could be further strengthened through formalized, structured counseling training programs. For instance, a study from Kenya (28) demonstrates that community engagement strengthens adherence to HIV care and prevention programs. In our study, several participants reported that the familiarity and reassurance provided by CBOs were instrumental in encouraging them to undergo screening. Moreover, confidentiality emerged as a key determinant of HIV testing uptake. Although a study from Uganda (39) reports that home-based testing can reduce stigma by offering a private setting, our findings offer a more nuanced this perspective. While some individuals perceived home testing as offering greater privacy and security, others expressed concerns about potential breaches of confidentiality and the risk of involuntary disclosure. These mixed perceptions underscore the need for tailored communication strategies and refined intervention approaches to strengthen confidentiality and improve acceptability.

The coordination role of the nongovernmental organization FMG further illustrates the complexity of implementing community-based strategies. FMG is responsible for training community counselors, facilitating their acceptance by health authorities, and, in some cases, directly managing the supply of testing materials. These results underscore the importance of integrating community-based strategies into formal health systems to enhance sustainability and effectiveness, as demonstrated in studies conducted in Uganda (40–42). Integrating community-based testing into Guinea’s national health policies could help address structural barriers and ensure long-term sustainability. Despite these advantages, several structural challenges persist. One major issue is the high turnover of community counselors, primarily due to insufficient financial incentives and a lack of continuous training. This observation is consistent with findings from rural South Africa (35) and studies conducted in five African countries (43), which emphasize that retaining community counselors remains a persistent challenge in resource-limited settings. To address these constraints, the literature recommends regular financial incentives and professional development opportunities as critical measures to sustain community-based interventions. Institutional reluctance also poses a significant barrier. Some health officials perceive that HIV testing conducted by non-medical community counselors does not meet biomedical standards, thereby limiting the integration of these actors into the national HIV response system. This finding aligns with evidence from South Africa (44), which indicates that institutional distrust toward non-medical personnel hinders the expansion of community-based testing. Similar resistance has been observed in other African contexts, where the lack of formal integration of community counselors slows intervention effectiveness (30, 45), further illustrating these operational challenges. Concerns about confidentiality, as previously discussed, further affect the acceptability of community-based HIV testing. Some participants reported fear of involuntary disclosure of their HIV status, echoing barriers identified in a study from Côte d’Ivoire (46). These mixed perceptions reveal the need for intensified awareness campaigns and the implementation of strict confidentiality protocols. Moreover, the effectiveness of community-based HIV testing is limited by external constraints, particularly frequent stockouts of testing kits and ARVs. As highlighted by a systematic review from sub-Saharan Africa (47), inefficient supply chains constitute a major barrier to the continuity of HIV services in resource-limited settings. Our findings indicate that such supply disruptions hinder community counselors’ performance and contribute to frustration among beneficiaries. Additionally, weak coordination between health facilities and CBOs results in operational inefficiencies. The absence of clear defined protocols and stakeholder roles exacerbates tensions and contributes to service fragmentation. A challenge is also documented in studies from rural Uganda, Kenya, and South Africa (33, 48). Finally, some local authorities oppose community-based HIV testing, arguing that delegating testing responsibilities to community counselors is inappropriate. This form of institutional reluctance is mirrored by findings from Ethiopia (47), where misalignment between community-based interventions and national health policies hinders effective implementation.

In light of these challenges, several key recommendations emerge for improving community-based HIV testing in Guinea. First, strengthening governance is essential. Developing clear operational protocols would enhance coordination between health centers and CBOs, facilitating better integration of community strategies into the formal health system. Second, optimizing the supply chain should be a strategic priority; establishing an efficient logistics system tailored to the needs of CBOs would help ensure a continuous supply of testing kits and ARV, thereby reducing stockouts. Moreover, continuous training for community counselors, alongside regular financial and non-financial incentives are critical to reducing workforce turnover and maintaining high-quality service delivery. Finally, reinforcing confidentiality safeguards and institutionalizing sub-recipients’ roles, such as FMG, would promote efficient technical management and foster harmonized stakeholder collaboration, ultimately enhancing both the program’s sustainability and impact.

### Study limitations

This study presents certain limitations that should be considered. First, the analysis primarily relies on participants’ perceptions, which may introduce response biases related to social desirability or the subjectivity of testimonies. Second, the deductive approach adopted did not allow for a comprehensive exploration of all sub-constructs within the CFIR framework, which may have limited the depth of contextual analysis. Lastly, the lack of comparison with other community-based HIV testing initiatives in different regions of Guinea reduces the transferability of the findings. However, several methodological strengths mitigate these limitations. The study reached data saturation, ensuring a comprehensive understanding of the acceptability and implementation challenges associated of community-based HIV testing. Furthermore, the use of the CFIR framework represents an innovative approach, providing one of the first systematic analyses of community-based HIV testing in Guinea through this theoretical perspective. This contributes to the external validity of the study. Lastly, data triangulation, incorporating insights from both service providers and beneficiaries, strengthened the internal validity of the findings, offering a robust and nuanced understanding of the intervention evaluated.

## Conclusion

The study highlights the complex dynamic of community-based HIV testing, an effective strategy for improving access to testing services, yet one that remains constrained by significant structural barriers. Among the most influential l facilitators identified, home-based testing was highly valued for its convenience, as it reduced both logistical and financial barriers. Additionally, the emotional and social support provided by community-based organizations (CBOs) played a pivotal role in fostering trust and encouraging participation in HIV testing. However, the study also identified several critical barriers that hinder the effectiveness of community-based HIV testing. These including frequent stockouts of testing kits and antiretrovirals, inadequate training and financial incentives for community counselors, persistent concerns about confidentiality, and insufficient coordination among stakeholders.

Addressing these barriers requires a multi-pronged approach, including strengthening supply chain management through real-time inventory tracking, providing continuous training and regular incentives for community counselors, and developing clear operational protocols to improve coordination between health facilities and CBOs. Furthermore, implementing strict confidentiality safeguards and fostering institutional trust in community-led interventions are essential for ensuring the long-term success of this approach.

Ultimately, this study provides a strategic roadmap for scaling up community-based HIV testing in Guinea and similar resource-limited settings, offering actionable insights to enhance both the effectiveness and sustainability of such interventions.

## Data Availability

The raw data supporting the conclusions of this article will be made available by the authors, without undue reservation.
